# Association between troponin-I levels and outcome in critically ill patients admitted to non-cardiac intensive care unit with high prevalence of cardiovascular risk factors

**DOI:** 10.1186/s12871-018-0515-7

**Published:** 2018-05-22

**Authors:** Abdulaleem Alatassi, Mohamad Habbal, Hani Tamim, Musharaf Sadat, Eman Al Qasim, Yaseen M. Arabi

**Affiliations:** 1 Intensive Care Department, King Saud bin Abdulaziz University for Health Sciences, King Abdullah International Medical Research Center, King Abdulaziz Medical City, Riyadh, Saudi Arabia; 2 Internal Medicine Department, University of Toronto, Toronto, Canada; 3 Internal Medicine Department, American University of Beirut Medical Center, Beirut, Lebanon; 4 King Saud bin Abdulaziz University for Health Sciences, King Abdullah International Medical Research Center, King Abdulaziz Medical City, Riyadh, Saudi Arabia

**Keywords:** Troponin-I, Critical care, ICU, Mortality

## Abstract

**Background:**

The association of troponin-I levels and outcome in medical-surgical ICU patients has been studied before in populations with low to moderate prevalence of cardiovascular risk factors. The objective in this article is to examine the association of troponin-I levels with hospital mortality in patients with high prevalence of cardiovascular risk factors who were admitted with medical-surgical indications to a non-cardiac intensive care unit.

**Methods:**

This was a retrospective study of adult patients admitted to a tertiary medical-surgical ICU between July 2001 and November 2011. Data were extracted from prospectively collected ICU and clinical laboratory databases. Patients were stratified based on the highest troponin-I level in the first 72 h of admission into four groups (*Group I* < 0.03, *Group II* = 0.03–0.3, *Group III* = 0.3–3 and *Group IV* > 3 ng/mL). Hospital mortality was the primary outcome. To study the association between elevated troponin-I and hospital mortality, we carried out multivariate logistic regression analyses with *Group I* as a reference group.

**Results:**

During the study period, 3368 patients had troponin-I levels measured in the first 72 h, of whom 1293 (38.3%) were diabetic and 1356 (40.2%) were chronically hypertensive. Among the study population, 2719 (81%) had elevated troponin-I levels (0.03 ng/mL and higher). Hospital mortality increased steadily as the troponin-I levels increased. Hospital mortality was 23.4% for Group I, 33.2% for Group II (adjusted odds ratio (aOR) 1.08, 95% confidence interval (CI) 0.84, 1.38), 49.6% for Group III (aOR = 1.64, 95% CI 1.24, 2.17), and 57.4% for Group IV (aOR 1.80, 95% CI 1.30, 2.49). The association of increased mortality with increased troponin level was observed whether patients had underlying advanced heart failure or not. Subgroup analysis showed an increased mortality in patients aged < 50 years, non-diabetics and not on vasopressors.

**Conclusion:**

In a population with high prevalence of diabetes and hypertension, elevated troponin-I was frequently observed in medical-surgical critically ill patients, and showed a level-dependent association with hospital mortality.

**Electronic supplementary material:**

The online version of this article (10.1186/s12871-018-0515-7) contains supplementary material, which is available to authorized users.

## Background

Troponin is a regulatory protein that controls calcium-mediated interaction of myosin and actin in the cardiac cells. Troponin-I elevation is an indicator of myocardial cell injury [1] and is considered the most specific biomarker for myocardial necrosis [2]. In 2000, the European Society of Cardiology and American College of Cardiology redefined myocardial infarction to include elevated troponin-I in appropriate clinical settings [3]. Although troponin-I elevation is used most often to determine if patients have had myocardial ischemia, there are other causes for troponin-I elevation in critically ill patients such as imbalance between oxygen supply and demand (e.g. sepsis, hypovolemic shock), right ventricular strain (e.g. pulmonary embolism) or inflammatory mediator-related myocardial cell injury [4]. Additionally, the release of troponin-I in the blood is associated with diseases that are not cardiac-related including end stage renal disease, subarachnoid hemorrhage, acute kidney injury, heart failure and myocarditis [5].

In patients with acute coronary syndromes, elevated troponin-I levels have been shown to be associated with a proportionately increasing risk of mortality, therefore troponin-I has been used by clinicians for the prognostication of such patients [6–8]. Troponin-I elevations are frequently seen in non-cardiac intensive care unit (ICU) patients and commonly occur due to conditions other than acute coronary syndromes [4, 9].

The prevalence of cardiovascular risk factors varies considerably around the globe. The incidence of diabetes in Saudi Arabia is very high (18.7%) vs. other Western countries (5–11%) [10]. According to the World Health Organization, in 2010–2013, the prevalence of hypertension in Canada was 19.5 while it was 29% in the USA, 30% in the UK, and 40.6% in Saudi Arabia [11, 12]. This high prevalence in the general population was reflected in our critically ill population in Saudi Arabia, which has a higher prevalence of diabetes and hypertension compared to most populations included in the systematic review [13].

Therefore, the aim of this study was to determine the association between elevated troponin-I and hospital mortality of medical-surgical critically ill patients in a population with high prevalence of cardiovascular risk factors and identify subgroups of critically ill patients with a particularly increased risk.

## Methods

### Setting

This was a retrospective cohort study of critically ill patients admitted between July 2001 and November 2011 in the general medical-surgical (non-cardiac) ICU at King Abdulaziz Medical City, Riyadh, Saudi Arabia. The study was approved by the Institutional Review Board of the Ministry of National Guard Health Affairs, Riyadh, Saudi Arabia. All patients above 18 years of age admitted to the ICU with troponin-I levels measured at least once during the first 72 h were included in the study. Patients with burns and brain death were excluded from the study. For patients who were admitted to the ICU more than once during the same hospitalization, we included the first admission only. Patients admitted under cardiology or cardiac surgery such as those with an acute ST-elevation myocardial infraction (STEMI, type I MI) and post-cardiac surgery were admitted to cardiac ICUs and were not included in the study. Troponin-I test ordering was based on the discretion of the treating ICU team.

### Data collection

Troponin-I was measured using Abbott ARCHITECT STAT Troponin-I Test, Abbot, Abbot Park, IL, USA. This troponin-I assay has a 10% coefficient of variation of 0.032 ng/mL (0.032 μg/L) according to the manufacturer. Data were extracted from prospectively collected ICU and clinical laboratory databases. The ICU has a database of all consecutive admissions with data being collected prospectively by a full-time data collector. The following variables were collected: age, gender, height, weight, acute physiology and chronic health evaluation (APACHE) II score [14], history of diabetes and chronic hypertension, chronic comorbidities (chronic liver disease, advanced heart failure, chronic respiratory disease, chronic renal disease and chronic immunosuppression) as defined by APACHE II system. Advanced heart failure is defined as New York Heart Association (NYHA) class IV heart failure. We grouped the main reasons for ICU admissions based on APACHE II system into the following categories: respiratory, cardiovascular, neurological, other medical, non-operative trauma and postoperative categories. The cardiovascular admission category includes admissions related to cardiovascular failure or insufficiency from hypertensive crisis, rhythm disturbances, acute decompensation of heart failure, hemorrhagic/hypovolemic shock, sepsis and dissecting thoracic/abdominal aneurysm. (Additional file 1: Table S1). Additionally, tachycardia (defined as heart rate > 150 beats/min) and hypotension (defined as systolic blood pressure (SBP) < 90 mmHg) in the first 24 h of ICU admission were documented. The following laboratory data were also documented during the ICU course: platelets, bilirubin, creatinine, lactate and INR. Moreover, acute kidney injury was defined according to Mortality Probability Model (MPM II) system by an absolute increase in serum creatinine of more than 176.8 μ /L (2.0 mg/dL) at any time in the first 24 h [15].

Patients were divided into four groups depending on the troponin-I level in the first 72 h Group *I* < 0.03, Group II = 0.03–0.3, Group III = 0.3–3 and Group IV > 3 ng/mL [16]. This categorization was carried out as per previously published studies.

### Outcomes

The primary outcome was hospital mortality. The secondary outcomes were ICU and hospital length of stay (LOS), mechanical ventilation duration, need for renal replacement therapy (RRT).

### Statistical analyses

Statistical analysis was performed using the Statistical Analysis Software (SAS, Release 8, SAS Institute Inc., Cary, NC, 1999, USA). Baseline characteristics, interventions and outcomes were reported as numbers and percentages for categorical variables and as means and standard deviations for continuous variables. They were compared among groups using Chi-square test and ANOVA, respectively.

To determine if troponin-I category was an independent predictor for hospital mortality, stepwise multivariate logistic regression analysis was used with Group I as the reference, where variables included in the model were those showing statistical significance or those known to be clinically relevant (age, APACHE II, sex, admission diagnosis, diabetes, chronic liver disease, chronic respiratory disease, chronic renal diseases, chronic immunosuppression, vasopressor use, sepsis, cardiac arrest, acute kidney injury, Glasgow Coma Scale, platelet, INR, bilirubin and lactic acid levels). Results were presented as aOR and 95% confidence intervals. Furthermore, we analyzed the data based on troponin-I measured in the first 24 h.

Lastly, we carried out subgroup analyses with stratification by the following variables: age, gender, sepsis, diabetes, vasopressor use, operative admission category, chronic cardiac, respiratory and liver disease, chronic immunosuppression, acute kidney injury, hypertension, and cardiac arrest, adjusting for the same clinically relevant covariates mentioned above. Tests of interactions to assess whether these variables are effect modifiers of the association between troponin-I and hospital mortality. A *p-value* of ≤.05 was considered statistically significant.

## Results

During the study period, 3368 patients out of 9238 patients had troponin-I levels measured of whom 1293 (38.3%) were diabetic and 1356 (40.2%) were chronically hypertensive. Among the study population, 2719 (81%) had elevated troponin-I (≥0.03 ng/mL). Of those, 1572 (47%) patients had troponin-I level between 0.03 and 0.3 (Group II), 750 (22%) between 0.3–3.0 (Group III) and 397 (12%) had troponin-I level > 3 ng/mL (Group IV). At baseline, the four groups had stepwise differences in several variables. Patients in Group I were the youngest (47 ± 21 years) and Group IV the oldest (age of 58 ± 20 years, *p* value< 0.0001). Similarly, APACHE II scores differed among the four groups (p value< 0.0001), being lowest in Group I (20 ± 8) and highest in Group IV (29 ± 9). The prevalence of diabetes and hypertension differed between the groups with the lowest being in Group I (27% diabetic and 30% hypertensive) and the highest in Group IV (52% diabetic and 51% hypertensive) (p value< 0.0001). Similar differences were seen with vasopressor use, the presence of sepsis, advanced heart failure, chronic renal disease, lactic acid, creatinine, and INR (Table 1).

**Table 1:** Baseline characteristics of the four troponin-I groups

Variables	Group I <0.03ng/ml *N* = 649	Group II 0.03 – 0.3ng/ml *N* = 1572	Group III 0.3 – 3ng/ml *N* = 750	Group IV >3ng/ml *N* = 397	*P*-value
Age, year, Mean ± SD	47.3 ± 21.1	55.2 ± 19.9	55.9 ± 20.6	58.4 ± 20.3	<0.0001
Gender, male, N (%)	429 (66.1)	991 (63.0)	478 (63.7)	267 (67.3)	0.31
APACHE II score, Mean ± SD	20.3 ± 8.6	23.0 ± 9	27.0 ± 9.3	29.7 ± 9.0	<0.0001
Glasgow Coma Scale, Mean ± SD	10.1 ± 4.3	10.1 ± 4.4	9 ± 4.7	8.0 ± 4.7	<0.0001
Admission diagnosis category, N (%)					
Respiratory	121 (18.6)	315 (20.0)	144 (19.2)	70 (17.6)	<0.0001
Cardiovascular^a^	127 (19.6)	451 (28.7)	289 (38.5)	202 (50.8)	
Neurologic	69 (10.6)	89 (5.7)	35 (4.7)	9 (2.3)	
Other medical	23 (3.5)	80 (5.1)	25 (3.3)	16 (4.0)	
Non-operative trauma	104 (16.0)	153 (9.7)	64 (8.5)	23 (5.8)	
Postoperative	205 (31.6)	484 (30.8)	193 (25.7)	77 (19.4)	
Admission category, N (%)					
Non-operative	444 (68.4)	1088 (69.2)	557 (74.3)	320 (80.6)	<0.0001
Postoperative	205 (31.7)	484 (30.8)	193 (25.7)	77 (19.4)	
Chronic co-morbidities, N (%)					
Chronic liver disease	60 (9.6)	196 (12.7)	93 (12.7)	37 (9.5)	0.08
Advanced heart failure	87 (13.8)	324 (20.9)	177 (24.1)	145 (37.1)	<0.0001
Chronic respiratory disease	84 (13.4)	231 (14.9)	120 (16.4)	55 (14.1)	0.44
Chronic renal disease	41 (6.5)	234 (15.1)	140 (19.1)	95 (24.2)	<0.0001
Chronic immunosuppression	64 (10.2)	195 (12.6)	67 (9.1)	31 (7.9)	0.01
Diabetes, N (%)	178 (27.4)	618 (39.3)	289 (38.5)	208 (52.4)	<0.0001
Chronic hypertension, N (%)	199 (30.7)	652 (41.5)	302 (40.3)	203 (51.1)	<0.0001
Mechanical ventilation, N (%)	477 (73.5)	1181 (75.1)	660 (88.0)	355 (89.4)	<0.0001
Vasopressor use, N (%)	213 (32.8)	677 (43.1)	445 (59.3)	266 (67.0)	<0.0001
Sepsis, N (%)	124 (19.1)	375 (23.9)	214 (28.5)	126 (31.7)	<0.0001
Admission post-cardiac arrest, N (%)	9 (1.4)	77 (4.9)	83 (11.1)	75 (18.9)	<0.0001
Admission physiologic characteristics					
Acute kidney injury, N (%)	51 (7.9)	232 (14.8)	182 (24.3)	107 (27)	<0.0001
Heart rate >150 beat/minute, Mean ± SD	0.02 ± 0.14	0.05 ± 0.21	0.06 ± 0.25	0.09 ± 0.29	<0.0001
Systolic Blood pressure <90 mmHg, Mean ± SD	0.20 ± 0.40	0.27 ± 0.45	0.39 ± 0.49	0.40 ± 0.49	<0.0001
Urine output (ml 1^st^ 24 hrs), Mean ± SD	2252.9 ± 1571.9	1877.6 ± 1415.7	1702.9 ± 1604.0	1422.4 ± 1301.3	<0.0001
PaO_2_/FiO_2_ <200, N (%)	204 (40.8)	598 (43.4)	360 (54.1)	187 (54.4)	<0.0001
Lab findings, Mean ± SD					
Platelet, 10^9^/L	257.3 ± 167.5	212.3 ± 142.7	186.9 ± 150.3	192.7 ± 136.4	<0.0001
Bilirubin, μmol/l	37.8 ± 87.5	55.2 ± 119.8	67.1 ± 132.7	44.4 ± 82.1	0.0002
Creatinine, μmol/l	108.4 ± 112.4	156 ± 149.3	192.2 ± 163.5	247.9 ± 198.3	<0.0001
Lactate, mg/dL	2.30 ± 2.30	3.02 ± 2.93	4.73 ± 4.20	5.70 ± 5.05	<0.0001
INR	1.35 ± 0.70	1.54 ± 0.87	1.79 ± 1.185	1.97 ± 1.35	<0.0001

*SD* standard deviation

^a^The cardiovascular admission category includes admissions related to cardiovascular failure or insufficiency from hypertensive crisis, rhythm disturbances, acute decompensation of heart failure, hemorrhagic/hypovolemic shock, sepsis and dissecting thoracic/abdominal aneurysm. Please refer to the Supplement for a complete list of admission categories

Hospital mortality increased steadily as the troponin-I levels increased (23% in Group I, 33% in Group II, 49% in Group III and 57% in Group IV). Multivariate analysis (with the following covariates: age, APACHE II, sex, admission diagnosis, diabetes, chronic liver disease, chronic respiratory disease, chronic renal diseases, chronic immunosuppression, vasopressor use, sepsis, cardiac arrest, acute kidney injury, Glasgow Coma Scale, platelet, INR, bilirubin and lactic acid levels) showed a level-dependent association between troponin-I levels and hospital mortality (adjusted OR (aOR) = 1.08, 95% CI = 0.84,1.38 for Group II, aOR = 1.64, 95% CI = 1.24, 2.17 for Group III and aOR = 1.80, 95% CI = 1.30, 2.49 for Group IV *(all in comparison with Group I)* (Table 2). Associations of Troponin-I elevations and ICU and hospital length of stay and duration of mechanical ventilation are shown in Table 2.

**Table 2:** Multivariate analysis of the four troponin groups and different clinical outcomes (with the following covariates: age, APACHE II, sex, admission diagnosis, diabetes, chronic liver disease, chronic respiratory disease, chronic renal diseases, chronic immunosuppression, vasopressor use, sepsis, cardiac arrest, acute kidney injury, Glasgow Coma Scale, platelet, INR, bilirubin and lactic acid levels)

Variables	Group I <0.03 ng/ml *N* = 649	Group II 0.03–0.3 ng/ml *N* = 1572	Group III 0.3– 3 ng/ml *N* = 750	Group IV >3 ng/ml *N* = 397	Group II vs Group I		Group III vs Group I		Group IV vs Group I	
Categorical variables					aOR (95% CI)	*P* value	aOR(95% CI)	*P* value	aOR (95% CI)	*P* value
Hospital mortality, N (%)	152 (23.4)	522 (33.2)	372 (49.6)	228 (57.4)	1.08 (0.84,1.38)	0.54	1.64 (1.24, 2.17)	0.0005	1.80 (1.30, 2.49)	0.0004
Continuous renal replacement therapy, N (%)	35 (5.4)	179 (11.4)	157 (20.9)	95 (23.9)	1.47 (0.95, 2 .26)	0.08	2.12 (1.35, 3.33)	0.001	1.99 (1.21, 3.27)	0.006
Hemodialysis, N (%)	24 (3.7)	144 (9.2)	86 (11.5)	51 (12.9)	1.73 (1.07, 2.79)	0.02	1.92 (1.15, 3.20)	0.01	1.85 (1.05, 3.26)	0.03
Continuous variables					Parameter estimate (95% CI)		Parameter estimate (95% CI)		Parameter estimate (95% CI)	
ICU LOS, days Mean ± SD	7.9 ± 9.1	8.8 ± 11	9.1 ± 10	8.4 ± 20.5	0.87 (-0.23,1.97)	0.12	1.13 (-0.13, 2.40)	0.08	0.48 (-1.02,1.98)	0.53
Hospital LOS, days Mean ± SD	54.8 ± 82.2	49.6 ± 107.4	40.7 ± 70.8	32.2 ± 182.8	-5.23 (-15.2, 4.7)	0.30	-14.18 (-25.6, 2.7)	0.02	-22.65 (-36.2, 9.1)	0.001
Mechanical ventilation duration, days, Mean ± SD	6.1 ± 8.3	7.3 ± 10.1	8.2 ± 10.1	8.6 ± 9.3	1.15 (0.26, 2.04)	0.011	2.07 (1.05, 3.09)	<0.0001	2.51 (1.30, 3.73)	<0.0001

*aOR* adjusted odds ratio

**Fig. 1:**
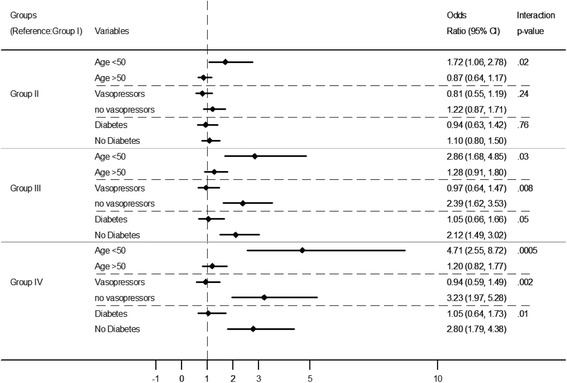
Association of troponin-I categories and hospital mortality in patients grouped by age, vasopressor use and history of diabetes in multivariate logistic regression analysis (with the following covariates: age, APACHE II, sex, admission diagnosis, diabetes, chronic liver disease, chronic respiratory disease, chronic renal diseases, chronic immunosuppression, vasopressor use, sepsis, cardiac arrest, acute kidney injury, Glasgow Coma Scale, platelet, INR, bilirubin and lactic acid levels). Results are presented as adjusted odds ratios and 95% confidence intervals

Subgroup analyses showed a significant association of troponin-I elevation with hospital mortality in age subgroups of < 50 years but not in those who were ≥ 50 years, (p for interaction = 0.0005 for Group IV). There was an association of troponin-I elevation with hospital mortality in patients who were on vasopressors but not those who were not on vasopressors (*p*-value for interaction = 0.0004 for Group IV). There was an incremental association of troponin-I levels with hospital mortality in non-diabetics but not in diabetics (p-value for interaction =0.01 for Group IV) (Table 3 and Fig. 1). The association between troponin-I levels and hospital mortality were not different (no effect modification) when patients were stratified by other variables including gender, sepsis, operative admission category, chronic cardiac disease, respiratory and liver disease, chronic immunosuppression, acute renal acute kidney injury, chronic hypertension and cardiac arrest (Table 3).

**Table 3:** Subgroup analysis for the association between different levels of troponin-I (all compared to Group I as a reference) and hospital mortality. The following variables were used as covariates in the model: age, APACHE II, sex, admission diagnosis, diabetes, chronic liver disease, chronic respiratory disease, chronic renal diseases, chronic immunosuppression, vasopressor use, sepsis, cardiac arrest, acute kidney injury, Glasgow Coma Scale, platelet, INR, bilirubin and lactic acid levels) Interaction test was performed for each subgroup

	Group II 0.03–0.3 ng/ml			Group III 0.3–3 ng/ml			Group IV >3 ng/ml		
Subgroups	aOR (95% CI)	*P* value	*P* valuefor interaction	aOR (95% CI)	*P* value	*P* value for interaction	aOR (95% CI)	*P* value	*P* value for interaction
Age									
< 50	1.72 (1.06, 2.78)	0.03	0.02	2.86 (1.68, 4.85)	0.0001	0.03	4.71 (2.55, 8.72)	<0.0001	0.0005
≥ 50	0.87 (0.64,1.17)	0.34		1.28 (0.91,1.80)	0.16		1.20 (0.82,1.77)	0.35	
Gender									
Male	0.99 (0.72,1.35)	0.95	0.50	1.68 (1.18, 2.39)	0.004	0.82	1.64 (1.10, 2.44)	0.02	0.63
Female	1.23 (0.82,1.86)	0.32		1.59 (1.00, 2.53)	0.05		2.16 (1.22, 3.81)	0.008	
Sepsis									
Yes	1.07 (0.66,1.74)	0.78	0.77	1.73 (1, 2.99)	0.05	0.95	1.69 (0.90, 3.17)	0.10	0.74
No	1.11 (0.83, 1.49)	0.47		1.68 (1.21, 2.33)	0.002		1.85 (1.26, 2.72)	0.002	
Vasopressors									
Yes	0.81 (0.55, 1.19)	0.28	0.24	0.97(0.64,1.47)	0.88	0.008	0.94 (0.59,1.49)	0.78	0.002
No	1.22 (0.87, 1.71)	0.24		2.39 (1.62, 3.53)	<0.0001		3.23 (1.97, 5.28)	<0.0001	
Admission category									
Non-operative	1.02 (0.77, 1.35)	0.91	0.53	1.48 (1.07, 2.03)	0.02	0.29	1.5 (1.03, 2.17)	0.03	0.07
Post-operative	1.47 (0.85, 2.55)	0.17		2.74 (1.48, 5.07)	0.001		3.29 (1.62, 6.71)	0.001	
Chronic liver disease									
Yes	0.79 (0.38, 1.64)	0.53	0.37	1.23 (0.51, 2.97)	0.65	0.52	3.19 (0.84,12.16)	0.09	0.4584
No	1.13 (0.87, 1.48)	0.36		1.74 (1.29, 2.34)	0.0003		1.82 (1.29, 2.56)	0.0006	
Advanced heart failure									
Yes	1.24 (0.71, 2.18)	0.45	0.63	2.29 (1.23, 4.26)	0.009	0.30	1.38 (0.73, 2.63)	0.32	0.23
No	1.04 (0.79,1.37)	0.79		1.52 (1.11, 2.09)	0.01		2.11 (1.43, 3.12)	0.0002	
Chronic respiratory disease									
Yes	0.76 (0.41, 1.42)	0.39	0.53	1.29 (0.65, 2.56)	0.47	0.64	1.2 (0.49, 2.95)	0.69	0.55
No	1.13 (0.86, 1.48)	0.39		1.70 (1.25, 2.32)	0.0007		1.85 (1.30, 2.63)	0.0006	
Chronic renal disease									
Yes	0.98 (0.44, 2.18)	0.96	0.94	2.27 (0.95, 5.42)	0.07	0.33	1.37 (0.55, 3.42)	0.50	0.45
No	1.09 (0.84, 1.41)	0.52		1.52 (1.13, 2.05)	0.006		1.94 (1.36, 2.75)	0.0002	
Chronic immunosuppression									
Yes	0.85 (0.45, 1.60)	0.61	0.19	1.49 (0.68, 3.28)	0.32	0.36	1.36 (0.49, 3.74)	0.55	0.37
No	1.11 (0.85, 1.46)	0.43		1.68 (1.24, 2.28)	0.0007		1.87 (1.32, 2.64)	0.0005	
Diabetes									
Yes	0.94 (0.63, 1.42)	0.77	0.76	1.05 (0.66,1.66)	0.84	0.05	1.05 (0.64,1.73)	0.84	0.01
No	1.1 (0.8,1.50)	0.57		2.12 (1.49, 3.02)	<0.0001		2.80 (1.79, 4.38)	<0.0001	
Acute Kidney injury									
Yes	1.02 (0.49, 2.12)	0.97	0.97	1.17 (0.54, 2.52)	0.70	0.23	1.27 (0.55, 2.95)	0.5811	0.33
No	1.08 (0.82,1.4)	0.59		1.8 (1.33, 2.44 )	0.0002		1.92 (1.34, 2.76)	0.0004	
Chronic hypertension									
Yes	1.08 (0.74,1.58)	0.70	0.64	1.4 (0.91, 2.15)	0.13	0.57	1.37 (0.85, 2.2)	0.20	0.15
No	1.01 (0.73,1.4)	0.96		1.79 (1.24, 2.59)	0.002		2.23 (1.41, 3.51)	0.0006	
Cardiac arrest									
Yes	4.21 (0.68, 26.19)	0.12	0.23	4.92 (0.79, 30.77)	0.09	0.50	6.48 (1.02, 41.23)	0.05	0.43
No	1.03 (0.80, 1.33)	0.81		1.62 (1.21, 2.16)	0.001		1.74 (1.23, 2.46)	0.002

When these analyses were repeated with using troponin-I measurements in the first 24 h (instead of 72 h), we found similar results, which are presented in Additional file 1: Tables S2, S3, S4.

## Discussion

In this study, we assessed the association between troponin-I levels and hospital mortality in medical-surgical critically ill patients with high prevalence of cardiovascular risk factors who were admitted to non-cardiac ICU. We found that 81% of these patients with troponin-I measurements had an elevated level. We found that troponin-I levels were independently associated with hospital mortality.

In a systematic review of 23 studies, Lim et al. assessed the frequency and association of elevated troponin-I with ICU mortality and length of stay and found a prevalence of troponin-I elevation of 43% (interquartile range 21–59%) [13]. The systematic review showed that patients with elevated troponin-I had an increased mortality by 2.5 folds (95% CI 1.9, 3.4, *P* < 0.001) and had an increased ICU stay by three days (95% CI 1, 5 days, *P* = 0.04) [13]. Most studies included in this review were conducted in countries with low to moderate prevalence of diabetes and hypertension.

Our findings are in accordance with the previously published systematic review [13]. However, the included studies in this review focused on specific diagnoses within the ICU, such as patients with sepsis, respiratory disease, acute coronary syndrome, or surgical patients [13]. Our study demonstrated that troponin-I elevation is associated with increased mortality in critically ill patients admitted to non-cardiac ICU regardless of patient population or admission diagnosis. Moreover, our population has high prevalence of cardiovascular risk factors; 38% of the patients had diabetes and 40% had hypertension. Out of the 23 studies included in the systematic review, only seven studies reported data on the prevalence of diabetes and hypertension. Five of those studies had a prevalence of diabetes ranging from 6 to 23%, and hypertension 16–31% [17–21]. Two small studies included in the Lim review had similar prevalence of diabetes with slightly higher prevalence of hypertension. The study by Landesberg et al., which included 101 critically ill patients, reported prevalence of 38% for diabetes and 58% for hypertension [22]. The study by Mehta et al., which included 37 patients, reported prevalence of 37% for diabetes and 43% for hypertension [23].

One marked difference between our study and the studies included in the systematic review was the proportion of patients found to have elevated troponin-I. Our study population had a higher proportion of patients with elevated troponin-I, 81% compared to a mean of 43% in the articles studied in the systematic review [13]. This difference can be accounted for by three main factors. First, troponin order was based on the discretion of the treating ICU team, which probably selected those at potential high risk of cardiac complications. This was reflected in the high proportion of patients with cardiovascular admitting diagnosis. Second, there was a higher prevalence of cardiovascular risk factors in our study population. Third, the lower cutoff limit for an elevated troponin-I level used in our study was 0.03 ng/ml as compared to the studies in the systematic review, which used a troponin-I range of 0.1–3.6 ng/ml [13]. Therefore, our study showed even if there is a small increase in troponin-I level it can have implications on mortality in critically ill patients.

Another difference is that Lim et al. systematic review suggested an association between troponin elevation and length of ICU stay, while our study did not demonstrate such an association [13].

Our study indicated a level-dependent association of troponin-I elevation with hospital mortality in patients who were less than 50 years of age, those not on vasopressors, and who were non-diabetics. These findings are surprising and may, at first, appear to be counterintuitive. Interestingly, these findings are consistent with studies that showed no association between troponin-I and mortality in patients admitted to ICU with sepsis and in elderly ill patients presenting to the Emergency Department [24, 25]. The observed increased mortality in patients who were less than 50 years of age and non-diabetics is consistent with the findings of Lim and Whitlock which showed that patients with elevated troponin levels for other than myocardial infarction had a worse outcome compared to those who did have myocardial infarction; with diabetes and old age being two of the most significant risk factors for myocardial infarction [16, 26]. Although our study did not distinguish between elevated troponin-I levels caused by myocardial infarction or due to other known causes of troponin-I elevation (e.g. sepsis, hypovolemic shock, pulmonary embolism or inflammatory mediator related myocardial cell injury), it is plausible that it takes a significant injury to the myocardium in patients with lower cardiovascular risk such as those who are young patients, not on vasopressors and non-diabetics, to cause troponin-I elevation compared to patients with higher cardiovascular risk factors such as older patients, on vasopressors and with diabetes [4]. This finding needs further validation in future studies. Based on current data, troponin measurement in critically ill patients may have prognostic value, but whether it can guide specific therapeutic interventions remains unknown and will need to be tested in randomized controlled trial.

The strengths of our study include the large sample size and the use of a prospectively collected ICU database. The limitations includes the retrospective design, being a single center study, utilizing troponin-I measurement instead of highly sensitive troponin (which was not available during the study period), unavailability of data on other risk factors (i.e. hyperlipidemia and smoking) because the data were obtained from an ICU database and unavailability of data regarding the specific causes of death. Although, troponin-I was ordered on a relatively large proportion of patients (36%), it was not obtained from the others, because routine troponin-I levels are not considered standard of care, and therefore the prevalence of high troponin-I cannot be generalized to all ICU patients. Nevertheless, this reflects real-life practice. Additionally, data on medications that may be cardioprotective were also not available. This latter effect was recently studied by Poe et al. who found that statins, β-blockers and aspirin have the potential to modify the mortality in patients with elevated cardiac troponin-I [27]. Notably, they showed that patients with no or intermediate elevation in cardiac troponin-I taking statins within 24 h of measurement had a lower mortality than those not taking statins. Furthermore, they showed that patients with high troponin-I elevation taking β-blockers and aspirin within 24 h of measurement had lower mortality than those not taking them.

Unlike patients with STEMI, who myocardial infarction due to an atherosclerotic plaque, [28] troponin-I elevation in general ICU patients is seldom related to obstructive coronary disease [1]. They usually experience Type II MI secondary to a variety of factors that increase mismatch between myocardial oxygen supply and demand; these include tachycardia, shock, vasopressors, inotropes, hypoxemia, anemia, and hypertension [27]. The mechanism of myocardium injury is important because most of data on MI treatment come from studies on Type I MI, and there are little data regarding the treatment of Type II MI. Our study is consistent with the VISION (Vascular Events in Noncardiac Surgery Patients Cohort Evaluation) study that included 15,000 patients, which showed that peak troponin-I measurement added incremental prognostic value to discriminate those likely to die within 30 days of non-cardiac surgery [29]. Although statins, β-blockers, and aspirin are often prescribed for ICU patients with troponin-I elevation, there are limited data regarding the effectiveness [27].

## Conclusion

Our study indicates that troponin-I elevation demonstrates a significant level-dependent association with hospital mortality in critically ill patients. As such, it may be used in conjunction with other measures as well clinical expertise to help clinicians prognosticating critically ill patients, as is currently done for troponin elevation in acute coronary syndromes [6]. Furthermore, we identified three subgroups of patients who were at increased risk of hospital mortality with troponin-I elevation: patients less than 50 years of age, non-diabetics and patients not on vasopressors. Further research needed to validate this differential association of troponin-I elevation and outcome by age, diabetes and vasopressor use. In addition, clinical trial to investigate the effect of therapeutic interventions in critically ill patients with troponin-I levels on outcomes.

## Additional file

Additional file 1: **Table S1.** Categories of main reasons of ICU admission. **Table S2.** Baseline characteristics of the four troponin-I groups based on troponin-I measured in the first 24 h. **Table S3.** Multivariate analysis of the four troponin-I groups and different clinical outcomes based on troponin-I measured in the first 24-h. **Table S4.** Subgroup analysis for the association between different levels of troponin-I measured in the first 24 h (all compared to Group I as a reference) and hospital mortality. The following variables were used as covariates in the model: age, APACHE II, sex, admission diagnosis, diabetes, chronic liver disease, chronic respiratory disease, chronic renal diseases, chronic immunosuppression, vasopressor use, sepsis, cardiac arrest, acute kidney injury, Glasgow Coma Scale, platelet, INR, bilirubin and lactic acid levels) Interaction test was performed for each subgroup. (DOCX 45 kb)

## Abbreviations

AKI: Acute Kidney Injury
aOR: Adjusted odds ratio
CI: Confidence interval
ICU: Intensive care unit
INR: International normalized ratio
LOS: Length of stay
MI: Myocardial infarction
RRT: Renal replacement therapy
