# Supplementary material for: Association between troponin-I levels and outcome in critically ill patients admitted to non-cardiac intensive care unit with high prevalence of cardiovascular risk factors
Source: BMC Anesthesiol. 2018 May 22;18:54. doi: 10.1186/s12871-018-0515-7 (PMC5964705; doi:10.1186/s12871-018-0515-7)
Supplement: Supplementary file 1 — Table S1. Categories of main reasons of ICU admission. Table S2. Baseline characteristics of the four troponin-I groups based on troponin-I measured in the first 24 h. Table S3. Multivariate analysis of the four troponin-I groups and different clinical outcomes based on troponin-I measured in the first 24-h. Table S4. Subgroup analysis for the association between different levels of troponin-I measured in the first 24 h (all compared to Group I as a reference) and hospital mortality. The following variables were used as covariates in the model: age, APACHE II, sex, admission diagnosis, diabetes, chronic liver disease, chronic respiratory disease, chronic renal diseases, chronic immunosuppression, vasopressor use, sepsis, cardiac arrest, acute kidney injury, Glasgow Coma Scale, platelet, INR, bilirubin and lactic acid levels) Interaction test was performed for each subgroup. (DOCX 45 kb) [file 12871_2018_515_MOESM1_ESM.docx]

**Additional file 1:**

**Table S1:** Categories of main reasons of ICU admission

| **Admission diagnosis categories** | **Diagnosis** |
| --- | --- |
| **Non-operative** |  |
| **Respiratory** |  |
| Respiratory failure or insufficiency from; | Asthma, allergy |
|  | Chronic obstructive pulmonary disease |
|  | Non-cardiogenic pulmonary edema |
|  | Post-respiratory arrest |
|  | Aspiration/poisoning/toxic |
|  | Pulmonary embolus |
|  | Infection |
|  | Neoplasm |
|  |  |
| **Cardiovascular** |  |
| Cardiovascular failure or insufficiency from: | Hypertension |
|  | Rhythm disturbances |
|  | Congestive heart failure |
|  | Hemorrhagic/hypovolemic shock |
|  | Coronary artery disease |
|  | Sepsis |
|  | Post cardiac arrest |
|  | Dissecting thoracic/abdominal aneurysm |
|  |  |
| **Neurology** | Seizure disorder |
|  | ICH/SDH/SAH |
|  |  |
| **Other medical** | Drug overdose |
|  | Diabetic ketoacidosis |
|  | GI bleeding |
|  |  |
| **Non-operative trauma** | Multiple trauma |
|  | Head trauma |
|  |  |
| **Post-operative** | Multiple trauma, head trauma |
|  | Admission due to Chronic cardiovascular disease |
|  | Peripheral vascular surgery, heart valve surgery |
|  | Craniotomy for neoplasm |
|  | Renal surgery for neoplasm |
|  | Renal transplant |
|  | Thoracic surgery for neoplasm |
|  | Craniotomy for ICH/SDH/SAH |
|  | Laminectomy and other spinal cord surgery |
|  | Hemorrhagic shock |
|  | GI bleeding |
|  | GI surgery for neoplasm |
|  | Respiratory insufficiency after surgery |
|  | GI obstruction or perforation |

**Table S2.**  Baseline characteristics of the four troponin-I groups based on troponin- I measured in the first 24 hours

| **Variables** | **Group I**  **<0.03ng/ml**  N = 636 | **Group II**  **0.03–0.3ng/ml**  N = 1457 | **Group III**  **0.3–3ng/ml**  N = 647 | **Group IV**  **>3ng/ml**  N = 321 | **P-value** |
| --- | --- | --- | --- | --- | --- |
| Age, years, Mean ±SD | 47.8±21.1 | 55.5±19.9 | 54.7±21.2 | 57.7±20.6 | <0.0001 |
| Gender, male, N (%) | 414 (65.0) | 912 (62.5) | 415 (64.1) | 218 (67.9) | 0.29 |
| APACHE II score, Mean ±SD | 20.5±8.7 | 23.2±9.1 | 26.9±9.4 | 29.2±9.0 | <0.0001 |
| Glasgow Coma Scale, Mean ±SD | 10.0±4.3 | 10.1±4.4 | 9±4.7 | 8.0±4.7 | <0.0001 |
| **Admission diagnosis category,** N (%) | | | |  |  |
| Respiratory | 121 (19.0) | 294 (20.1) | 120 (18.5) | 53 (16.5) | <0.0001 |
| Cardiac | 133 (20.9) | 416 (28.5) | 251 (38.7) | 165 (51.4) |  |
| Neurology | 66 (10.3) | 84 (5.7) | 28 (4.3) | 9 (2.8) |  |
| Other medical | 23 (3.6) | 77 (5.2) | 20 (3.0) | 12 (3.7) |  |
| Non-operative trauma | 96 (15.0) | 134 (9.2) | 58 (8.9) | 21 (6.5) |  |
| Post-operative | 197 (31.6) | 452 (31.2) | 170 (26.2) | 61 (19.0) |  |
| **Admission category**, N (%) | | | |  |  |
| Non-operative | 439 (69.0) | 1005 (68.9) | 477 (73.7) | 260 (81.0) | <0.0001 |
| Post-operative | 197 (30.9) | 452 (31.0) | 170 (26.2) | 61 (19.0) |  |
| **Chronic co-morbidities**, N (%) | | | |  |  |
| Chronic liver disease | 66 (10.6) | 180 (12.5) | 79 (12.5) | 24 (7.5) | 0.06 |
| Chronic cardiovascular disease | 84 (13.5) | 309 (21.5) | 154 (24.3) | 116 (36.4) | <0.0001 |
| Chronic respiratory disease | 88 (14.2) | 201 (14.0) | 107 (16.9) | 47 (14.8) | 0.35 |
| Chronic renal disease | 45 (7.2) | 226 (15.7) | 118 (18.6) | 75 (23.5) | <0.0001 |
| Chronic immunosuppression | 65 (10.5) | 185 (12.9) | 53 (8.3) | 22 (6.9) | 0.001 |
| Diabetes, N (%) | 178 (27.4) | 581 (39.8) | 246 (38.0) | 172 (53.5) | <0.0001 |
| Hypertension, N (%) | 195 (30.6) | 611 (41.9) | 260 (40.1) | 168 (52.3) | <0.0001 |
| Mechanical ventilation, N (%) | 477 (73.5) | 1181 (75.1) | 660 (88.0) | 355 (89.4) | <0.0001 |
| Vasopressor use, N (%) | 211 (33.1) | 626 (42.9) | 376 (58.1) | 219 (68.2) | <0.0001 |
| Sepsis, N (%) | 126 (19.8) | 350 (24.0) | 178 (27.5) | 94 (29.2) | <0.0001 |
| Admission post cardiac arrest, N (%) | 11 (1.7) | 70 (4.8) | 72 (11.1) | 67 (20.8) | <0.0001 |
| **Admission physiologic characteristics** | | | | | |
| Acute kidney injury, N (%) | 53 (8.3) | 225 (15.4) | 151 (23.3) | 82 (25.5) | <0.0001 |
| Heart rate >150 beat/minute, Mean ±SD | 0.02±0.14 | 0.05±0.21 | 0.06±0.25 | 0.09±0.29 | <0.0001 |
| Systolic Blood pressure <90 mmHg, Mean ±SD | 0.20±0.40 | 0.27±0.45 | 0.39±0.49 | 0.40±0.49 | <0.0001 |
| Urine output (ml 1^st^ 24 hrs), Mean ±SD | 2185.4±1562.4 | 1854.9±1441.9 | 1720.7±1570.3 | 1478.5±1334.3 | <0.0001 |
| PaO_2_/FiO_2_ <200, N (%) | 203 (41.4) | 549 (43.0) | 305 (54.1) | 150 (53.7) | <0.0001 |
| **Lab findings,** Mean ±SD | | | | | |
| Platelet, 10^9^/L | 257.3±174.0 | 211.9±142.0 | 185.3±147.4 | 191.2±129.8 | <0.0001 |
| Bilirubin, µmol/l | 41.8±96.8 | 55.5±119.2 | 63.2±132.0 | 36.5±62.7 | 0.0016 |
| Creatinine, µmol/l | 111±110.7 | 159.7±148.6 | 188.1±165.8 | 247.6±194.3 | <0.0001 |
| Lactate, mg/dL | 2.37±2.48 | 3.07±3.00 | 4.89±4.47 | 5.45±4.81 | <0.0001 |
| INR | 1.37±0.73 | 1.55±0.91 | 1.77±1.18 | 1.93±1.30 | <0.0001 |

SD= standard deviation.

**Table S3.** Multivariate analysis of the four troponin groups and different clinical outcomes based on troponin-I measured in the first 24-hours

| **Variables** | **Group I**  **<0.03ng/ml**  N= 649 | **Group II**  **0.03 – 0.3ng/ml**  N= 1572 | **Group III**  **0.3 – 3ng/ml**  N= 750 | **Group IV**  **>3ng/ml**  N= 397 | **Group II vs Group I** | | **Group III vs Group I** | | **Group IV vs Group I** | |
| --- | --- | --- | --- | --- | --- | --- | --- | --- | --- | --- |
| **Categorical variables** | | | | | **aOR (95%CI)** | **P value** | **aOR(95%CI)** | **P value** | **aOR (95%CI)** | **P value** |
| Hospital mortality, N (%) | 158 (24.8) | 505 (9.3) | 318 (94.1) | 169 (52.6) | 1.60 (1.30, 1.97) | <0.0001 | 2.92 (2.30, 3.70) | <0.0001 | 1.80 (1.30, 2.49) | 0.0004 |
| Continuous renal replacement therapy, N (%) | 41 (6.4) | 175 (12.0) | 129 (19.9) | 70 (21.8) | 1.98 (1.39, 2.82) | 0.0002 | 3.61 (2.49, 5.23) | <0.0001 | 4.5 (2.67, 6.11) | <0.0001 |
| Hemodialysis, N (%) | 24 (3.7) | 136 (9.3) | 73 (11.2) | 39 (12.1) | 2.62 (1.68, 4.1) | <0.0001 | 3.24 (2.01, 5.21) | <0.0001 | 3.52 (2.08, 5.97) | <0.0001 |
|  | | | | | | | | | | |
| **Continuous variables** |  |  |  |  | **Parameter estimate (95% CI)** |  | **Parameter estimate (95% CI)** |  | **Parameter estimate (95% CI)** |  |
| ICU LOS, days, Mean ±SD | 7.9±9.2 | 8.5±10 | 9.1±10.2 | 8.4±22.1 | 0.53 (-0.58, 1.64) | 0.34 | 1.02 (-0.28, 2.33) | 0.12 | -0.10 (-1.70, 1.50) | 0.90 |
| Hospital LOS, days, Mean ±SD | 54.8±81.4 | 47.8±107.4 | 40.4±73.5 | 32.7±202.1 | -6.12 (-16.4, 4.22) | 0.24 | -13.4 (-25.6,-1.31) | 0.02 | -21.2 (-36.1,- 6.35) | 0.005 |
| Mechanical ventilation duration, days, Mean ±SD | 6.1±8.5 | 7.0±10.1 | 8.2±10.5 | 8.2±8.7 | 0.91 (0.008, 1.81) | 0.04 | 2.12 (1.06, 3.18) | <0.0001 | 2.11 (0.81, 3.41) | 0.0014 |

aOR = adjusted odds ratio. The following variables were adjusted for: age, APACHE II, sex, admission diagnosis, diabetes, chronic liver, chronic respiratory, chronic renal diseases, and chronic immunosuppression, vasopressor use, sepsis, cardiac arrest, acute kidney injury, Glasgow coma scale, platelet, INR, bilirubin and lactic acid levels, MPM: mortality prediction model

| **Table S4.** Subgroup analysis for the association between different levels of troponin I measured in the first 24 hours (all compared to Group I as a reference) and hospital mortality. The following variables were used as covariates in the model: age, APACHE II, sex, admission diagnosis, diabetes, chronic liver disease, chronic respiratory disease, chronic renal diseases, chronic immunosuppression, vasopressor use, sepsis, cardiac arrest, acute kidney injury, Glasgow Coma Scale, platelet, INR, bilirubin and lactic acid levels) Interaction test was performed for each subgroup. | | | | | | | | | |
| --- | --- | --- | --- | --- | --- | --- | --- | --- | --- |
|  | **Group II**  **0.03-0.3ng/ml** | | | **Group III**  **0.3 -3ng/ml** | | | **Group IV**  **>3ng/ml** | | |
|  | aOR (95% CI) | P value | P value  for interaction | aOR (95% CI) | P value | P value  for interaction | aOR (95% CI) | P value | P value  for interaction |
| Age | | | | | | | | | |
| < 50 | 1.64 (1.01, 2.64) | 0.04 | 0.04 | 2.46 (1.44, 4.20) | 0.0010 | 0.06 | 3.07 (1.61, 5.84) | 0.0006 | 0.002 |
| ≥ 50 | 0.92 (0.68, 1.24) | 0.60 |  | 1.28 (0.90, 1.83) | 0.15 |  | 0.95 (0.63, 1.44) | 0.82 |  |
| Gender | | | | | | | | | |
| Male | 0.99 (0.72, 1.36) | 0.96 | 0.34 | 1.87 (1.30, 2.69) | 0.0006 | 0.16 | 1.36 (0.89, 2.07) | 0.14 | 0.80 |
| Female | 1.28 (0.85, 1.93) | 0.23 |  | 1.20 (0.74, 1.95) | 0.43 |  | 1.35 (0.74, 2.48) | 0.32 |  |
| Sepsis | | | | | | | | | |
| Yes | 1.07 (0.66, 1.75) | 0.76 | 0.73 | 1.82 (1.02, 3.22) | 0.039 | 0.64 | 1.10 (0.56, 2.14) | 0.77 | 0.46 |
| No | 1.17 (0.87, 1.56) | 0.28 |  | 1.64 (1.18, 2.29) | 0.003 |  | 1.55 (1.04, 2.32) | 0.029 |  |
| Vasopressors | | | | | | | | | |
| Yes | 0.78 (0.52, 1.15) | 0.21 | 0.12 | 0.95 (0.61, 1.45) | 0.81 | 0.02 | 0.70 (0.43, 1.14) | 0.16 | 0.003 |
| No | 1.32 (0.94, 1.86) | 0.10 |  | 2.22 (1.48, 3.32) | <0.0001 |  | 2.47 (1.44, 4.23) | 0.001 |  |
| Admission category | | | | | | | | | |
| Non-operative | 1.05 (0.79, 1.40) | 0.71 | 0.47 | 1.54 (1.11, 2.14) | 0.008 | 0.53 | 1.19 (0.81, 1.76) | 0.36 | 0.03 |
| Post-operative | 1.5 (0.86, 2.58) | 0.15 |  | 1.99 (1.06, 3.75) | 0.03 |  | 2.38 (1.12, 5.07) | 0.02 |  |
| Chronic liver disease | | | | | | | | | |
| Yes | 0.77 (0.37, 1.60) | 0.48 | 0.37 | 0.93 (0.37, 1.60) | 0.87 | 0.44 | 2.05 (0.49, 8.60) | 0.32 | 0.61 |
| No | 1.17 (0.89, 1.53) | 0.24 |  | 1.72 (1.26, 2.34) | 0.0005 |  | 1.40 (0.97, 2.02) | 0.06 |  |
| Advanced heart failure | | | | | | | | | |
| Yes | 1.38 (0.77, 2.48) | 0.26 | 0.33 | 2.37 (1.24, 4.52) | 0.008 | 0.26 | 1.098 (0.55, 2.19) | 0.78 | 0.30 |
| No | 1.03 (0.78, 1.36) | 0.81 |  | 1.47 (1.06, 2.04) | 0.01 |  | 1.57 (1.04, 2.38) | 0.03 |  |
| Chronic respiratory disease | | | | | | | | | |
| Yes | 0.67 (0.35, 1.27) | 0.22 | 0.32 | 1.16 (0.57, 2.36) | 0.66 | 0.51 | 0.82 (0.32, 2.11) | 0.69 | 0.42 |
| No | 1.18 (0.90, 1.56) | 0.21 |  | 1.67 (1.21, 2.29) | 0.001 |  | 1.41 (0.97, 2.06) | 0.06 |  |
| Chronic renal disease | | | | | | | | | |
| Yes | 1.30 (0.60, 2.78) | 0.49 | 0.55 | 2.56 (1.09, 6.02) | 0.03 | 0.14 | 1.33 (0.54, 3.26) | 0.53 | 0.69 |
| No | 1.09 (0.84, 1.42) | 0.52 |  | 1.46 (1.08, 1.99) | 0.014 |  | 1.46 (1.0, 2.13) | 0.04 |  |
| Chronic immunosuppression | | | | | | | | | |
| Yes | 0.92 (0.47, 1.77) | 0.80 | 0.13 | 1.68 (0.69, 4.06) | 0.24 | 0.53 | 0.98 (0.32, 2.99) | 0.97 | 0.33 |
| No | 1.15 (0.87, 1.51) | 0.31 |  | 1.61 (1.18, 2.20) | 0.002 |  | 1.39 (0.96, 2.01) | 0.07 |  |
| Diabetes | | | | | | | | | |
| Yes | 1.03 (0.68, 1.56) | 0.86 | 0.88 | 1.09 (0.67, 1.75) | 0.72 | 0.11 | 0.91 (0.54, 1.53) | 0.72 | 0.06 |
| No | 1.08 0.79, 1.50) | 0.60 |  | 2.02 (1.40, 1.50) | 0.0001 |  | 1.95 (1.21, 3.15) | 0.006 |  |
| Acute renal failure | | | | | | | | | |
| Yes | 1.0 (0.50, 2.07) | 0.99 | 0.79 | 1.32 (0.60, 2.89) | 0.48 | 0.49 | 1.0 (0.41, 2.40) | 0.99 | 0.34 |
| No | 1.10 (0.84, 1.44) | 0.48 |  | 1.62 (0.84, 1.44 ) | 0.002 |  | 1.40 (0.95, 2.05) | 0.08 |  |
| Chronic hypertension | | | | | | | | | |
| Yes | 1.10 (0.74, 1.61) | 0.62 | 0.07 | 1.4 (0.91, 2.21) | 0.11 | 0.82 | 1.07 (0.64, 1.76) | 0.79 | 0.18 |
| No | 1.06 (0.76, 1.48) | 0.69 |  | 1.67 (1.13, 2.45) | 0.008 |  | 1.67 (1.03, 2.72) | 0.03 |  |
| Cardiac arrest | | | | | | | | | |
| Yes | 3.36 (0.70 , 16.15) | 0.12 | 0.30 | 2.99 (0.62, 14.38) | 0.17 | 0.70 | 3.66 (0.74, 17.89) | 0.10 | 0.53 |
| No | 1.06 (0.82 , 1.37) | 0.62 |  | 1.58 (1.17, 2.13) | 0.002 |  | 1.27 (0.87, 1.84) | 0.20 |  |

aOR = adjusted odds ratio. The following variables were adjusted for: age, APACHE II, sex, admission diagnosis, diabetes, chronic liver, chronic respiratory, chronic renal diseases, and chronic immunosuppression, vasopressor use, sepsis, cardiac arrest, acute kidney injury, Glasgow coma scale, platelet, INR, bilirubin and lactic acid levels.
